# The effect of the heat used during composite processing on the mechanical properties of fibrous reinforcement of polypropylene-based single-polymer composites

**DOI:** 10.1038/s41598-022-24764-8

**Published:** 2022-11-28

**Authors:** Tamás Bárány, Bálint Morlin, László Mihály Vas

**Affiliations:** Department of Polymer Engineering, Faculty of Mechanical Engineering, Budapest University of Technology and Economics, Műegyetem rkp. 3., Budapest, H-1111 Hungary

**Keywords:** Composites, Polymers

## Abstract

In this study, we investigated the effect of heat treatment on the mechanical properties of high-tenacity polypropylene (PP) fibers. An application field of versatile polypropylene as fibers and tapes is the reinforcement of single-polymer composites. During consolidation at an elevated temperature, typically near the melt temperature of PP, the heat causes molecular relaxation of the strongly oriented molecular chains, which impairs mechanical properties. We investigated the shrinkage of PP single fibers isothermally and anisothermally, and heat-treated PP single fibers and multifilament rovings in a temperature range of 120–190 °C for 5–20 min in a constrained and an unconstrained arrangement. The heat-treated fibers and rovings were then tensile tested and their residual mechanical properties were determined and compared to the as-received rovings. We analyzed the tensile characteristics mathematically, applying the statistical fiber-bundle-cell modeling method, and described the measured and averaged stress–strain curves with fitted E-bundles having fibers with nonlinear tensile characteristics. The tensile modulus of the constrained fibers treated for 5 min decreased less in the whole heat treatment temperature range but considerably decreased further with increasing treatment time. Conversely, their tensile strength decreased only slightly, and treatment time had a minor effect up to 180–190 °C (above the melting temperature of the fiber). The results proved that constraining is a useful tool for preserving the reinforcing ability of high-tenacity polymer fibers.

## Introduction

A class of polymer composites is single-polymer composites. They have been attracting considerable interest for decades due to their excellent mechanical properties, low density, and ease of reprocessability^1,2^. The reinforcement and the matrix of a single-polymer composite are both polymers^1^. When they have the same chemical composition, they can be defined as single-polymer (SPC) or self-reinforced polymer composites (SRPC) (e.g. single polypropylene or self-reinforced polypropylene composites). If the chemical composition of the components slightly differs (e.g. either or both components contain some co-monomers (less than 15–20 wt%)), the composites are called all-polymer composites or polymer-based single-polymer composites (e.g., polypropylene-based single-polymer or all-polypropylene composites). Self-reinforced polymeric materials have been produced for a long time since they can be produced by shaping, forming, or stretching thermoplastic polymers. With suitable technology, the molecular chains can be oriented, resulting in an anisotropic structure, i.e., much higher mechanical strength and modulus in the drawing direction. In this case, the self-reinforced structure can be produced in-situ during processing or ex-situ after processing, by changing the molecular conformation without any additional reinforcement. It is used in many applications: film hinges, shrink wrap, packing strips, synthetic polymer fibers, etc.

The concept of single polymer composites (SPCs) was introduced by Capiati and Porter in 1975 using the example of high-density polyethylene (HDPE). They called such composites one-polymer composites^3^. These composites are prepared from highly oriented, highly drawn, high-tenacity polymer pre-products (fiber, tape, or the textile made thereof). Single polymer composites are consolidated at an elevated temperature from the reinforcement itself (one constituent)^4–7^, or from two constituents from the same polymer^8–11^, or from slightly different polymers (e.g. the matrix is a copolymer and differs only in co-monomer content from the reinforcement)^12–15^. The reinforcement used is also a self-reinforced material since it shows anisotropy due to the orientation of molecular chains caused by a high stretching ratio. In the composites, the high-strength, high-modulus fibers and tapes act as reinforcement, while the low-strength embedding material acts as the matrix. For reinforcement, semi-crystalline thermoplastic polymers are used most commonly because of their high drawability and the attainable strength and modulus increment compared to amorphous polymers. For the matrix, however, either an amorphous or a semi-crystalline thermoplastic can be used. The exhaustive characterization of reinforcing fibers and tapes is very important since this constituent determines the strength and modulus of the resulting composite. Polypropylene (PP) is a viscoelastic thermoplastic polymer material; its mechanical properties are highly time- and temperature-dependent in the range of the processing temperature, so the basic study of PP reinforcement is not sufficient, whereas it is enough in the case of traditional reinforcements (e.g. glass fiber, basalt fiber, carbon fiber). The time-dependent mechanical behavior of PP can be characterized with a stress-relaxation test. The stress in question can be measured on the macrostructural level of the fibers, but this phenomenon is the result of microstructural relaxation processes of a stochastic nature at the molecular level of the PP. The decrease in macroscale stress is the result of the local stress reduction of the coiled molecule segments caused by debonding secondary bonds, uncoiling and straightening, and on the other hand by the irreversible displacement of the center of gravity of the molecule, when its environment allows it; its new position will be fixed by new secondary bonds. The relaxation time of the molecule segments as a time constant determines the rate of local stress reduction and a shorter relaxation time means faster relaxation. Consequently, the set of the relaxation time values characteristic of the material determines the rate of the stress reduction of the PP fiber. The relaxation time values strongly depend on the temperature—with increasing temperature, relaxation time decreases, thus the local relaxation processes become faster and more intensive. When the fibers are not strained and the temperature increases, the molecule segments in the oriented fibers attempt to take up their most probable conformation at the higher temperature, which means that the segments straightened to a certain degree will coil again. These micro-processes result in fiber shrinking. When the fibers are strained, shrinking is hindered, which may increase the stress in the fibers.

The high-tenacity polymer fibers have a highly oriented molecular structure due to the intense unidirectional drawing during processing. This oriented molecular structure is thermodynamically unstable, so the heat applied during the consolidation of composites causes molecular relaxation, i.e. decreasing orientation since the molecular chains attempt to reverse to a random coil conformation (to an isotropic structure). The decreasing orientation decreases the tensile strength and modulus of the fibers and thereby affects the mechanical properties of the composites. Generally, SPCs are consolidated slightly below the melting temperature of the reinforcement, therefore molecular relaxation impairs mechanical properties considerably. Besides the processing temperature, relaxation is also influenced by geometrical constraints (e.g., we kept the fibers fixed) and mechanical stresses (tension, compression). The free relaxation of fibers is much greater than the relaxation of constrained fibers. Composites are consolidated under pressure (typically 5–10 MPa), which impedes molecular relaxation and shrinkage due to fiber–fiber or tape–tape and fiber/tape–matrix friction under pressure.

The unconstrained, drawn polymer fibers have a somewhat higher melting temperature (e.g., 6–8 °C higher for PP^10^) than the bulk material. According to the Gibbs free energy formula, the melting temperature (T_m_) is given by the ratio of the enthalpy (ΔH) and the entropy difference (ΔS) between crystal and liquid^16^ (1):

1 $$T_{m} = \frac{\Delta H}{{\Delta S}}.$$

ΔH is affected by the interaction forces between the molecular chains. Irregularity in the chains (e.g.co-monomer), small crystals and crystal imperfections result in smaller ΔH, and consequently, lower T_m_. Drawn and longer molecular chains (typically for drawn polymer fibers) give larger interaction forces that increase ΔH and thereby T_m_. ΔS is determined by the conformational possibilities of the molecular chains^16^. If a polymer has an oriented molecular structure, the polymer tries to set back its isotropic structure upon heating, and relaxation and shrinkage occur. If relaxation is hampered (e.g. the fibers are fixed and their length cannot change), ΔS remains small, and T_m_ is higher. This phenomenon is called the overheating effect. Barkoula et al.^16^ studied the overheating effect for apolar (polyethylene and PP) and polar (polyamide 6 and polyethylene terephthalate) polymer fibers prepared with a home-built lab-scale melt-spinning device and concluded that the overheating effect is smaller for polar (8–10 °C) than apolar (20–25 °C) polymers. They produced PP fibers with different post-drawing temperatures and draw ratios and pointed out that the post-drawing temperature is the most beneficial around 140 °C and a draw ratio below 10 did not affect the morphology, thereby the melting temperature. At a higher draw ratio, λ = 14, there was a shift of 10 °C if the fiber was constrained.

Single-polymer composites are typically consolidated by compression molding under pressure. Pressure has a similar but slighter impact; it hinders the relaxation of the highly-drawn fibers, and acts as a constraint during processing. Furthermore, the pressure decreases the mobility of the oriented molecular chains, so ΔS decreases and T_m_ increases.

The mechanical behavior of polymer composites is characterized by tensile test data, which can be supplemented by modeling results. The classical fiber bundle models (FBMs)^17,18^ have rather widely been used in physics to describe, for example, the percolation threshold phenomenon or transitions of phases or states in the materials science to model the damage and failure processes^19,20^. The fiber-bundle-cells (FBC) method uses a set of different idealized statistical fiber bundles (fiber-bundle-cells denoted by E, EH, ES and ET) as building elements of a model network and it has been developed mainly for applications in the polymer materials science, including textile-based structures^21–24^. The simplest FBC model is a single E-bundle that is used in this present study where the model fibers have a nonlinear stress–strain relationship, and the expected tensile force of the model can be fitted to the measured tensile force. The results may add information to the direct test data and provide a basis for further analysis. In general, the determination of the expected tensile force needs the calculation of a parametrized integral; however, in the case of an E-bundle, it is just an analytical formula instead of an integral.

In this paper, we studied the effect of heat treatment (varying time and temperature) on the mechanical properties of constrained and unconstrained high-tenacity polypropylene fibers and rovings.

## Materials and methods

### Materials

We used a highly oriented polypropylene multifilament with a linear density of 3300 dtex (Stradom S.A., Czestochowa, Poland). On the basis of 50 optical microscopy measurements, the average diameter of the single fibers and the standard deviation were about 34.6 µm ± 1.4 µm. A multifilament or roving contains 400 single fibers. The melting temperature of the polymer is 171 °C, based on the DSC test results (10 °C/min, first heating run).

### Heat treatment of the fibers

**Figure 1:**
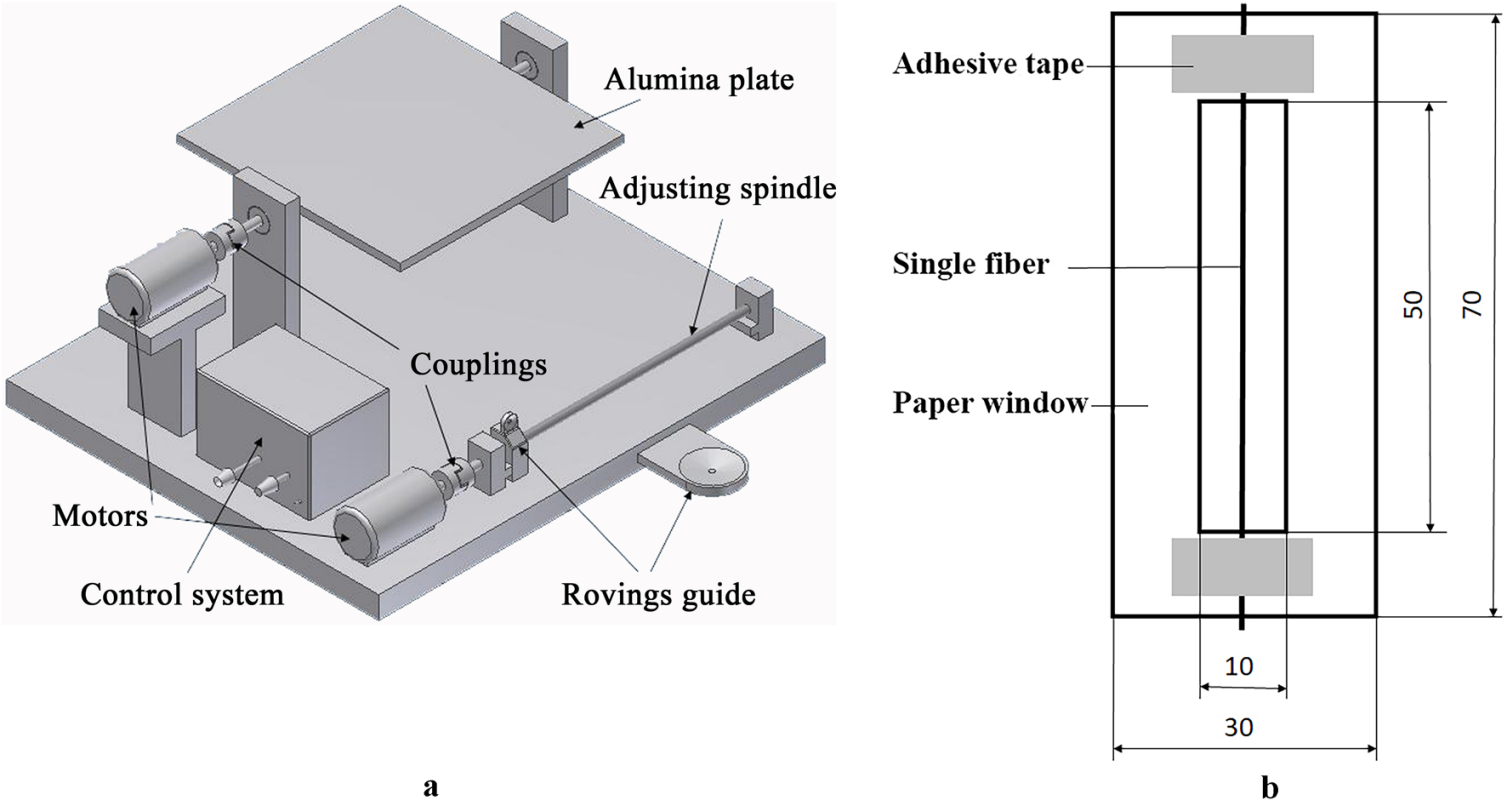
Winding unit (**a**) and fiber preparation (**b**).

Before performing mechanical tests on the fibers, we heat-treated them (i) freely (unconstrained) and (ii) wound onto an aluminum (Al) plate (constrained) with a home-made winding unit (Fig. 1a). Before winding, we fixed one end of the fiber on the Al plate with an adhesive; then we placed 20 parallel rovings next to each other by evenly rotating the plates. The unwinding of the roving from the bobbin and the friction of the roving on the yarn guides kept the roving constantly tight (ca. 0.3 cN/dtex which is in accordance with ASTM D3822-07^25^). After winding, we fixed the other end of the multifilament and removed it and the Al plate from the winding unit.

Both the free rovings (laid on an Al plate without their ends fixed) and the wound rovings with the Al plate were heat-treated in a temperature-controlled heat chamber (UT6, Heraeus Holding GmbH, Hanau, Germany). The treating temperatures were 120, 140, 160, 180, and 190 °C, and treatment time was 0, 5, 10, 15 and 20 min. We removed the rovings from the Al plate after cooling them down, and measured the length of the freely relaxed rovings and determined shrinkage.

As references, we applied free and wound roving samples conditioned at ambient temperature in the laboratory (23 °C) as well as single fibers taken from these rovings.

### Mechanical characterization of the fibers and rovings

We performed tensile tests on the rovings and the single fibers taken off the rovings. In the case of single fibers, we prepared them on paper windows (Fig. 1b). After preparation, we determined the diameter of each fiber with an Olympus BX51M light microscope (Olympus GmbH Hamburg, Germany). Tensile tests were performed on a Zwick Z005 universal testing machine (Zwick GmbH, Ulm, Germany) installed with a 20 N load cell (sensitivity: 0.0001 N), with a crosshead speed of 5 mm/min, a preload of 0.01 N, and a clamping length of 50 mm. In each case, we tested at least 10 single fibers, but for reference and close to the melting temperature of the fiber (140 and 160 °C), we tested at least 50 single fibers. From the tensile curves, we determined the tensile modulus (between the strain of 0.0015 and 0.0035, where the curve is straight), tensile strength (at the maximal force), and elongation at break. In the case of rovings, we did the same. We used a 5 kN load cell (sensitivity: 0.01 N) and a preload of 0.01 N. When calculating the tensile strength of the rovings, we used the average single fiber diameter measured after the same heat treatment, multiplied by the number of fibers in the roving (400 fibers).

We performed dynamic mechanical analysis (DMA Q800, TA Instruments, Delaware, USA) on the single fibers in tensile mode with a clamping length of 20 mm and a heating rate of 3 °C/min. We used different load settings:

- For the relaxation test, a single fiber was tensioned with a constant 2 MPa (stress level: 0.3%), and the change in fiber length was registered in the temperature range of 0–180 °C. This low level of load (0.2% of the maximal load) and the short duration of the test makes creep negligible;
- For the isothermal relaxation test, a single fiber was tensioned with a constant 2 MPa at 50, 80, 100, 120, 130, 140, 150, 160, 165 and 170 °C, and the change in fiber length was registered. After the relaxation tests, the fibers were cooled to room temperature and immediately tensile tested with a loading rate of 0.5 N/min with the help of the DMA;
- The DMA test was performed in the temperature range of − 50 to 160 °C, with a frequency of 1 Hz and deformation of 0.1%.

## Results and discussion

### Relaxation tests of single fibers in DMA

**Figure 2:**
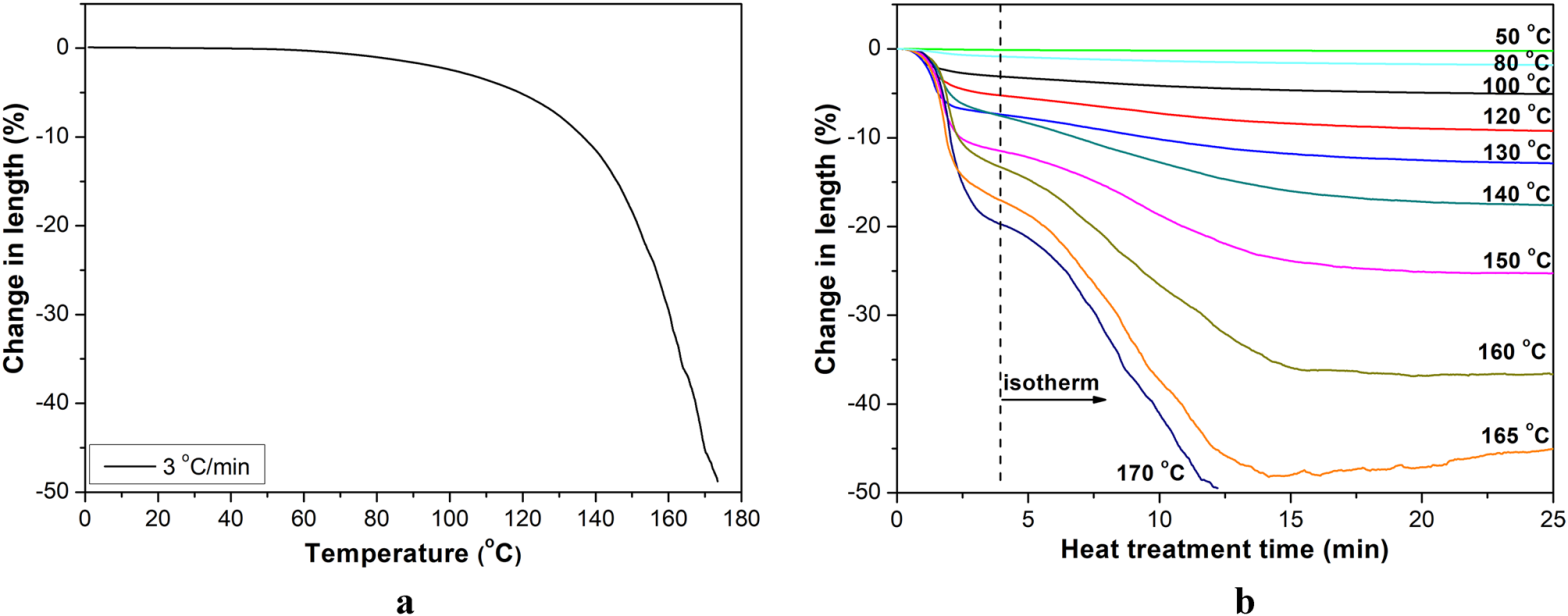
Relaxation tests of single fibers in DMA: Change in length as a function of temperature (**a**) and treating time at constant temperatures (**b**).

Figure 2 shows the results of the relaxation tests performed by DMA. For an accurate determination of shrinkage caused by relaxation, the single fiber was loaded slightly (2 MPa—0.3% of its tensile strength), and the deformation was registered during heating up to 180 °C. Shrinkage increased with increasing temperature (Fig. 2a); at 120 °C, the length decreased by 5%, while at 160 °C, it decreased by 30%. Figure 2b shows the isothermal curves as a function of time for different temperatures. Shrinkage became more significant near the melting temperature of the fibers (171 °C), and constantly increased at a given temperature but saturated after ca. 15 min at different values, depending on treatment temperature. With increasing temperature, the residual tensile strength of the relaxed single fiber also dropped from 505 MPa (relaxed at 100 °C) to 200 MPa (relaxed at 160 °C).

**Figure 3:**
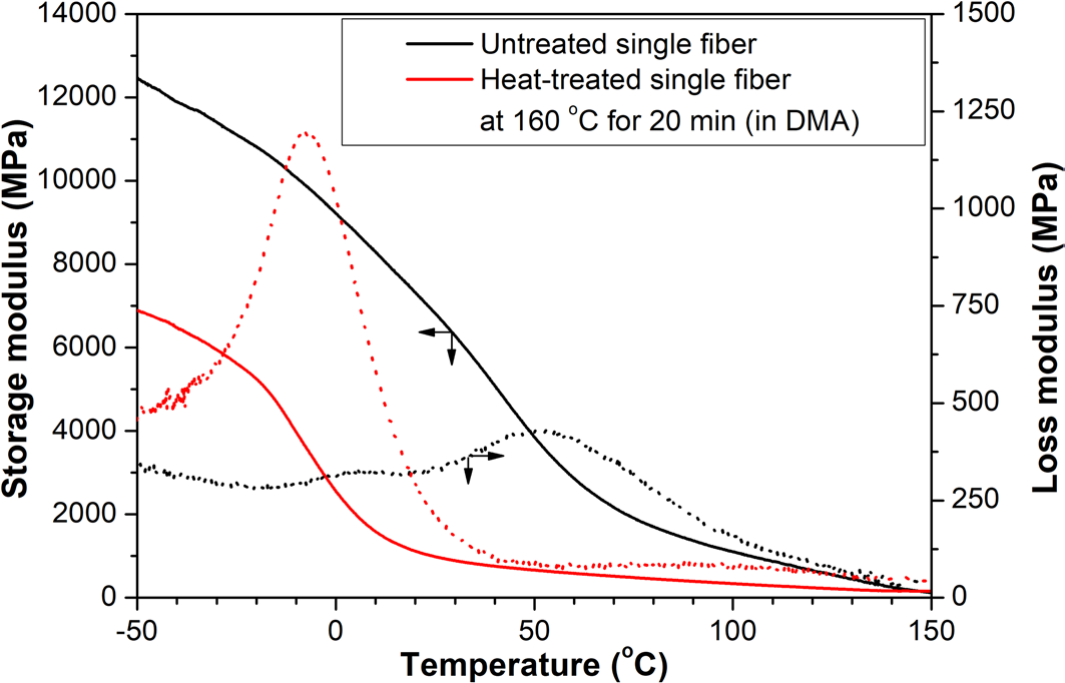
Storage and loss modulus of the untreated and the heat-treated (160 °C, 20 min) single fibers.

To determine the effect of heat treatment on the mechanical properties of single PP fibers, we examined the untreated and the heat-treated fibers (160 °C, 20 min) by DMA. Figure 3 shows their storage modulus. The storage modulus decreased in the tested temperature range, and the heat treatment caused a significant decrement, too.

### The effect of heat treatment on the tensile mechanical properties of single fibers and rovings

The results of the DMA tests showed that it is crucial to know the impact of the treatment temperature and time and the constraint applied to the relaxation process and the resulting properties of the reinforcing fibers. Therefore, we studied in detail the mechanical properties of the single fibers and rovings in a temperature and time range in a free and a fixed, constrained (wound) state. The rovings were heat-treated in a temperature chamber free (taken from the bobbin) and fixed (wound on an alumina plate) for a treatment time between 0 and 20 min and at a treatment temperature between 120 and 190 °C. After heat treatment, the single fibers (taken from the rovings) and the rovings were tensile tested.

**Figure 4:**
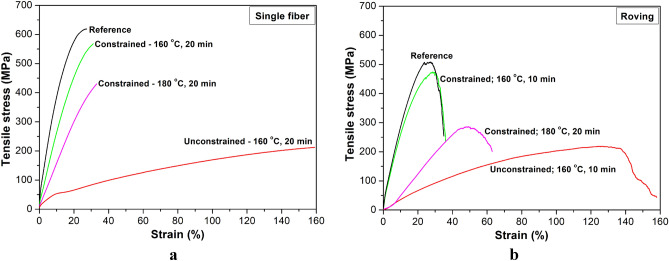
Typical stress–strain curves of single fibers (**a**) and rovings (**b**) for untreated and heat-treated fibers (**b**) at different treatment settings.

Figure 4a shows the typical stress–strain curves of untreated and treated single fibers. In the case of wound samples, where shrinkage is greatly reduced, the curves are similar to the reference curve but with a decreasing gradient (modulus) and a slightly increasing strain at break if the treatment temperature increases. Single fibers from freely heat-treated rovings show a significant drop in tensile strength and modulus and several times higher strain at break value. The rovings behaved similarly (Fig. 4b).

We measured the diameter of the single fibers and determined its change due to shrinkage. Higher shrinkage leads to a larger diameter (the diameter of the original fiber is 34.6 µm ± 1.4 µm). In the case of wound samples, the diameter only increased a few percent, but freely treated fibers showed an increment of 10–15% (as a function of treatment time; treated at 140 °C) and 35–40% (as a function of treatment time; treated at 160 °C). The lengths of the rovings were also measured; there was no measurable shortening in wound rovings, but there was a significant length decrease for freely heat-treated rovings (ca. 30% for 140 °C, 20 min, and 50% for 160 °C, 20 min).

**Figure 5:**
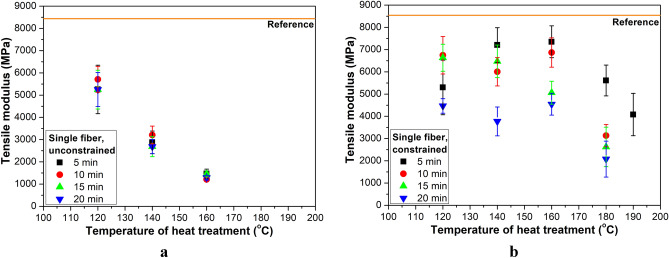
Tensile modulus of single fibers as a function of heat treatment temperature for different treatment times: in the case of free (**a**) and constrained (**b**) treatment.

Figure 5 shows the tensile modulus of the single fibers. The significant relaxation and shrinkage due to free heat treatment caused a remarkable decrement in tensile modulus. In this case, relaxation is a fast process. Therefore, there is no notable decrease after 5 min at the same treatment temperature. For constrained fibers, the tensile modulus does not change significantly in the temperature range of 120 to 160 °C for a given treatment time. At a given temperature, increasing treatment time decreased the tensile modulus. At 180 °C (above the melting temperature of the unconstrained highly oriented fibers), the modulus dropped and further reduced with increasing heat treatment time. The fibers treated at 190 °C for 5 min were suitable for testing, and the modulus further decreased. In the longer heat treatment at 190 °C, the fibers welded together and made the single fiber test impossible. It shows good agreement with the findings of Barkoula et al. They pointed out that the melting temperature peak of a constrained overdrawn multifilament can reach 195 °C^15^.

**Figure 6:**
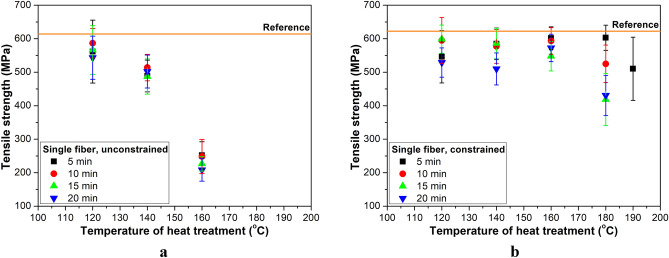
Tensile strength of single fibers as a function of heat treatment temperature for different treatment times: in the case of free (**a**) and constrained (**b**) treatment.

The tensile strength of freely heat-treated fibers (Fig. 6a) is affected similarly to the tensile modulus as a function of temperature, and treatment time caused a similarly negligible change. In the case of constrained heat treatment (Fig. 6b), tensile strength did not change as a function of treatment temperature up to 180 °C (for 5 min) and 160 °C (for 10–20 min). At 180 °C, time had a more considerable effect than at lower temperatures. At 190 °C, a treatment time of 5 min only caused a ca 15% decrease in tensile strength.

The tensile modulus of freely treated fibers decreased more considerably (30–40%) even at 120 °C than the tensile modulus of constrained fibers (15–20%), compared to the reference value. Tensile strength showed a slight decrease (5–10%) for fibers treated freely at 120 °C for 5 min and treated constrained at 120–180 °C for 5 min, compared to the reference value. Increasing treatment time caused a further decrease in tensile strength. Below the melting temperature of the fiber, more than 50% of the reference modulus and more than 65% of the reference strength can be restored even with 20 min of heat treatment.

**Figure 7:**
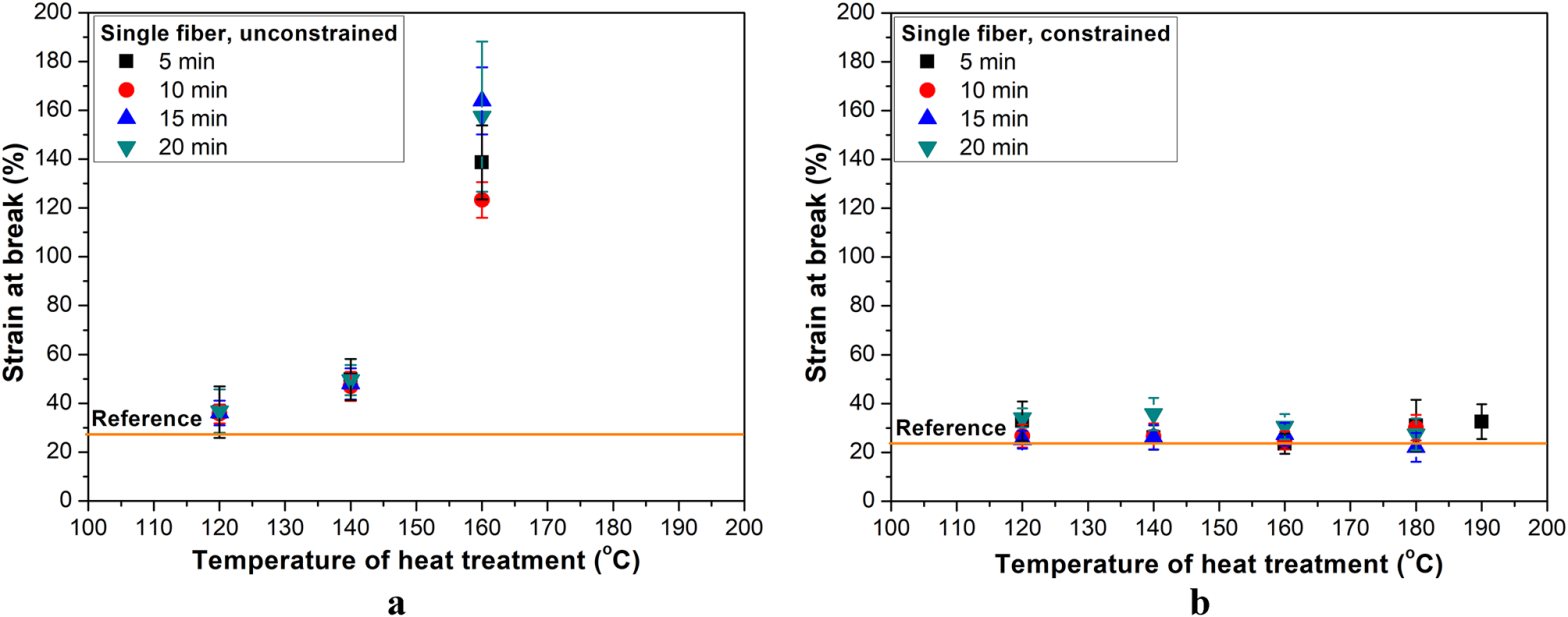
Strain at break of single fibers as a function of heat treatment temperature for different treatment times: in the case of free (**a**) and constrained (**b**) treatment.

Figure 7 depicts strain at break. In the case of unconstrained relaxation (Fig. 7a), for shorter treatment times and for constrained treatment for the whole tested temperature range, strain at break increased only slightly. Significantly decreasing molecular orientation leads to much larger strain at break values, as in the case of fibers treated freely at 160 °C^26^.

**Figure 8:**
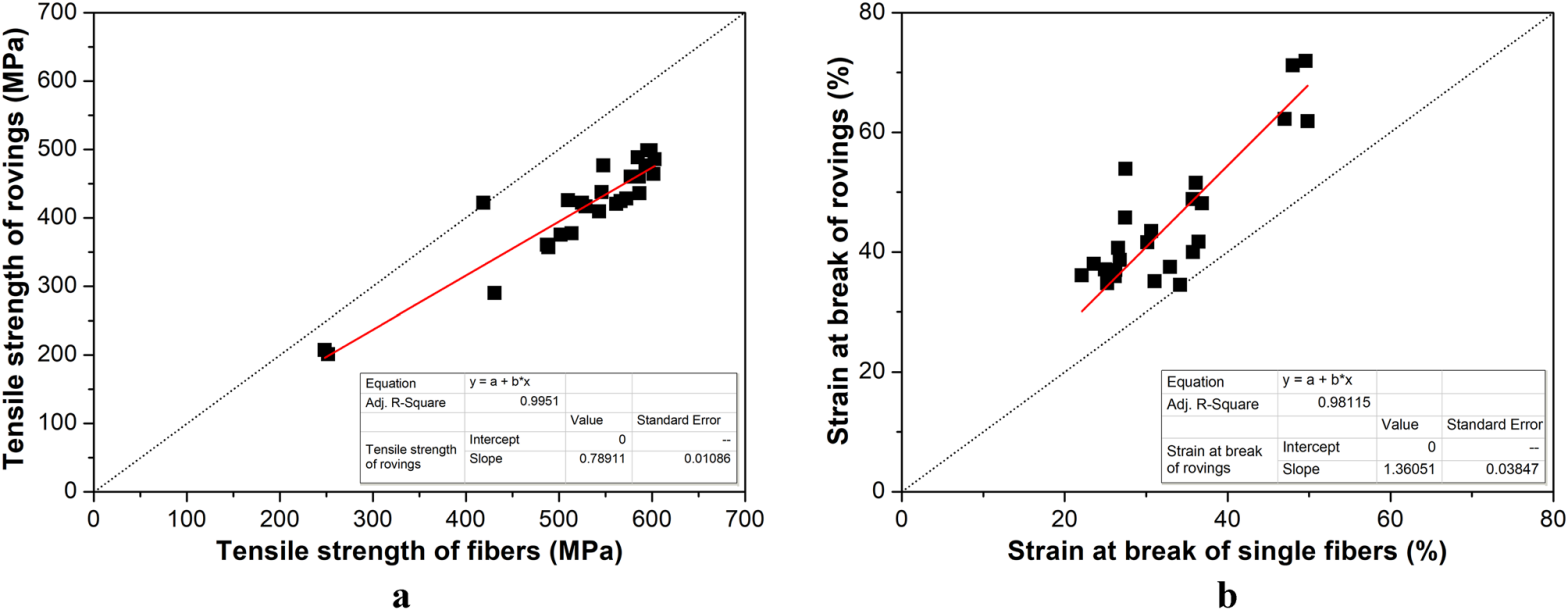
Correlation between the mechanical properties determined on single fibers (x axis) and rovings (y axis): in the case of tensile strength (**a**) and strain at break (**b**).

We also tensile tested multifilaments (rovings). With the testing of rovings, the collective behavior of the single constituent fibers can also be studied. The heat treatment caused similar changes in the tensile modulus, strength, and strain at break, as in the case of single fibers. A comparison of tensile strength (Fig. 8a) and strain at break (Fig. 8b) of the roving and the related single fibers shows that the single fibers provide better strength but smaller deformability than the rovings, which consist of 400 single fibers.

### Modeling of tensile mechanical properties of single fibers and rovings

To analyze the statistical bundle behavior of the fibers and rovings, we applied the simplest fiber-bundle-cell (FBC)^21–24^, the nonlinear E-bundle^21^. It has fibers with nonlinear tensile characteristics, k(x), which is the relationship between the tensile strain (x = ε) and the tensile force (F) in the fibers.

#### The fitted nonlinear tensile characteristics of the fibers and rovings

**Figure 9:**
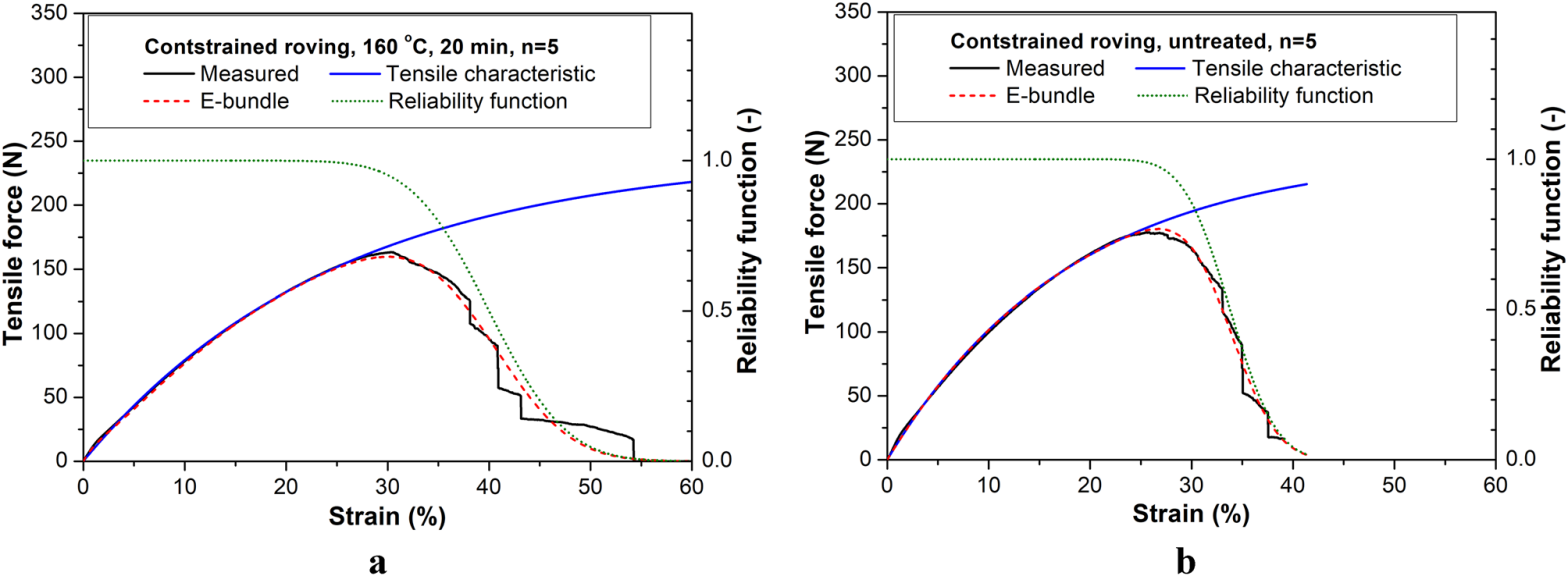
Measured mean tensile test curve, the fitted tensile characteristic, reliability function, and nonlinear E-bundle curve of constrained fibers (**a**) and rovings (**b**) without heat treatment.

**Figure 10:**
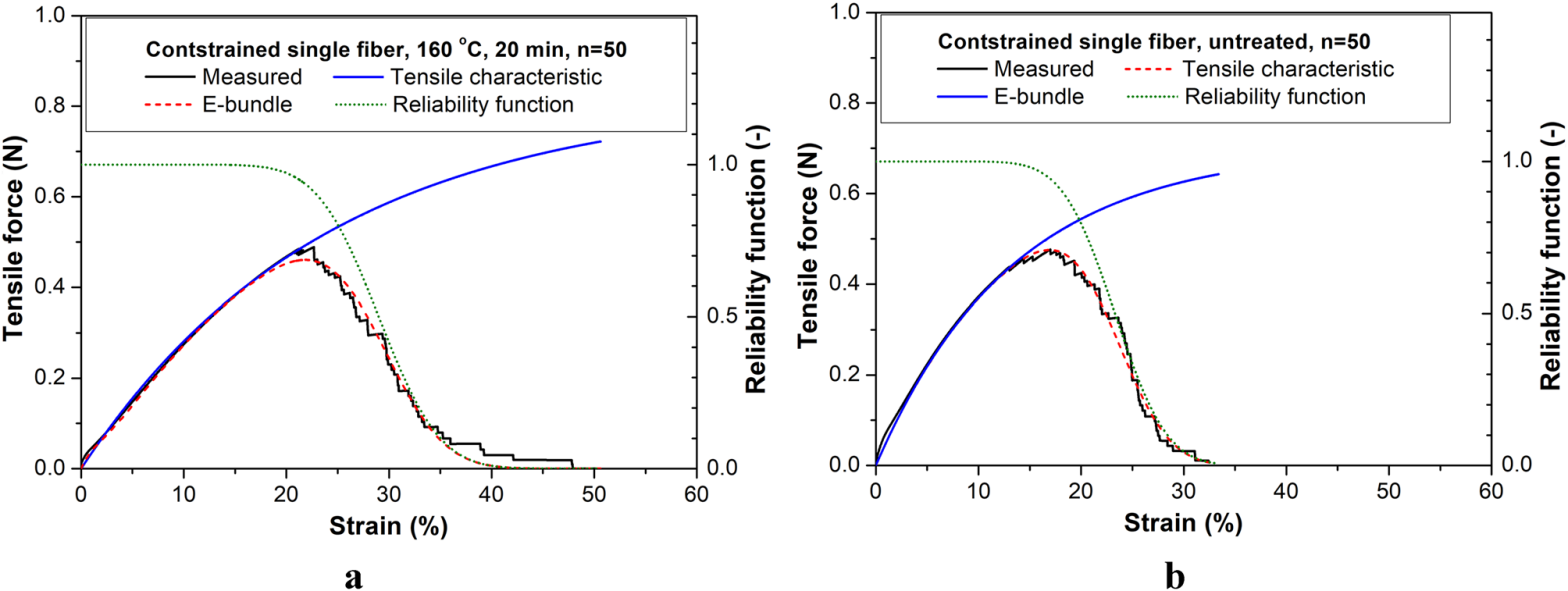
Measured mean tensile test curve, and the fitted tensile characteristic, reliability function, and nonlinear E-bundle curve of constrained fibers (**a**) and rovings (**b**), with heat treatment (160 °C, 20 min).

Based on the tensile tests, the mathematical form of the tensile characteristics describing the undamaged force vs. strain function applicable for both the untreated (Fig. 9) and heat-treated (Fig. 10) fibers and rovings with a good approximation is the convex linear combination of two exponential functions (k_0_ and k_A_). Functions k_0_(x) and k_A_(x) describe the initial behavior and the asymptotic behavior at large deformations, respectively (x ≥ 0) (2):

2 $$k\left( x \right) = k_{0} \left( x \right)w\left( x \right) + k_{A} \left( x \right)\left( {1 - w\left( x \right)} \right) = a_{0} \left( {1 - e^{{ - b_{0} x}} } \right)a_{E} e^{{ - b_{E} x}} + a_{A} \left( {1 - e^{{ - b_{A} x}} } \right)\left( {1 - a_{E} e^{{ - b_{E} x}} } \right)$$

where (*a*_*0*_, *b*_*0*_), (*a*_*A*_, *b*_*A*_), and (*a*_*E*_, *b*_*E*_) are the parameters of the initial and the large deformation asymptotes and the exponential weighting function, w(x), respectively (subsequently *a*_*E*_ = 0 or 1). Equation (2) corresponds to the tensile test response of two Maxwell models connected in parallel.

In the case of tensile tests of fibers or yarns, Hooke’s law has often been used in form of F = Kε, where F is the tensile force, ε is the strain, and K = EA is the tensile stiffness where E is the tensile modulus and A is the area of the cross section. Thus, the unit of tensile stiffness (N) is the same as that of tensile force. Its use is advantageous when the cross-sectional area (A) of fibers is of a significantly statistical nature and/or hard to measure. Regarding Eq. (2), the initial tensile stiffness is determined by the first term (3) (a_E_ = 1, *b*_*E*_ = 0):

3 $$K = K_{0} = a_{0} b_{0}$$

When *a*_*E*_ = 0, then only the second term shows up (4):

4 $$k\left( x \right) = a_{A} \left( {1 - e^{{ - b_{A} x}} } \right)$$

Experience shows that in the case of the untreated samples, Eq. (4) can also be used well. This corresponds to the case of the negligible first term (*b*_*E*_ = ∞). In the latter case, the slope of the initial tangent, that is, the initial tensile stiffness, is given by (5):

5 $$K = K_{A} = a_{A} b_{A}$$

For large x values, the function described by Eq. (2) tends asymptotically to constant *a*_*A*_.

#### Expected tensile force of the nonlinear E-bundle applied

In the case of the nonlinear E-bundle, that is, the simplest fiber-bundle-cell, the expected tensile force can be given in a product form (x ≥ 0) (6)^21,22,24^:

6 $$E\left( {F\left( x \right)} \right) = \overline{F}\left( x \right) = k\left( x \right)\left( {1 - Q_{{\varepsilon_{S} }} \left( x \right)} \right) = k\left( x \right)R\left( x \right)$$

where Q_εS_(x) is the distribution function of the fiber breaking strain, ε_S_, while its complement is the reliability function of the fiber bundle, denoted by R(x). In Eq. (6), the tensile characteristic, k(x) given by Eq. (2), determines the relationship between the controlled bundle strain (x = ε) and the force response when there is no damage. The reliability function of the bundle, R(x) = 1 − Q_εS_(x), describes the damage and failure process of the E-bundle, and represents the proportion of the fibers intact at the strain level given by x = ε. The tensile tests of fiber and roving samples were performed at a constant strain rate, thus, failure was characterized by breaking strain ε_S_. Its distribution function gives the probability that the sample breaks at the strain load x = ε, that is, Q_εS_(x) = P(ε_S_<x). Regarding the E-bundle as the model of the samples, Q_εS_(x) represents the proportion of the broken fibers at this strain level. At a very low strain load, the probability of fiber breakage is close to zero (Q_εS_(x) ≈ 0), and at the same time, reliability is close to 1 (R(x) ≈ 1). In this case, the tensile force vs. strain relationship is approximately ideal, meaning that F(x) = k(x)R(x) ≈ k(x). At a higher strain load, the probability of fiber breakage cannot be neglected. Consequently, the resistance against load decreases because of the damage and failure, therefore F(x) = k(x)R(x) < k(x). Hence, when the parameters of k(x) and R(x) are known—for example, from fitting to the measured mean tensile force vs. strain relationship as it was in our case—the fitted functions F(x) = k(x)R(x) can be used for deeper analysis of the tensile tests and the effects of heat treatment. Moreover, the fitted functions enable the creation of a material model for composites reinforced by the fibers tested and modeled as well.

While the peak coordinates of the expected force of the fitted E-bundle are in strong relation to those of the measured force peaks, some other characteristic properties can be obtained from the FBC model only.

In this study, the distribution function of the fiber breaking strain could be described with normal distribution with expected value E(ε_S_) = ${\overline{\varepsilon }}_{S}$ (MBS − Mean Breaking Strain) and standard deviation D(ε_S_) (SD). The coefficient of variation (CV) is their ratio.

Besides the MBS and SD of the fiber breaking strain, that is the breaking strain of the model fiber elements of the fitted E-bundle, some additional characteristic parameters determined from the fitted FBC model are as follows:

1. Peak point coordinates of the bundle curve (7):
  7 $$F^{*} = \overline{F}\left( {\varepsilon^{*} } \right)$$
2. Inflection point coordinates on the descendent part of the bundle curve (8):
  8 $$F_{inf} = \overline{F}\left( {\varepsilon_{inf} } \right)$$
3. The strain value belonging to the 5% peak force on the descendant part of the bundle curve (9):
  9 $$0.05F^{*} = \overline{F}\left( {\varepsilon_{0.05} } \right)$$
4. The utilization factor of fiber strength for the bundle (FBC model) (10), which is a kind of effectiveness considering the overall utilization of the building elements that are the fibers in any fibrous material tested^21–23^:
  10 $$FH^{*} = \frac{{F^{*} }}{{k\left( {\overline{\varepsilon }_{S} } \right)}}$$
5. The relative mean squared error of fitting (11):
  11 $$RMSE = \frac{1}{{F_{max} }}\sqrt {\frac{1}{M}\mathop \sum \limits_{i = 1}^{M} \left( {\overline{F}\left( {x_{i} } \right) - F_{measured} \left( {x_{i} } \right)} \right)^{2} }$$
  where F_max_ is the measured maximum tensile force, and M is the number of sampled data.

#### Results of FBC modeling

**Figure 11:**
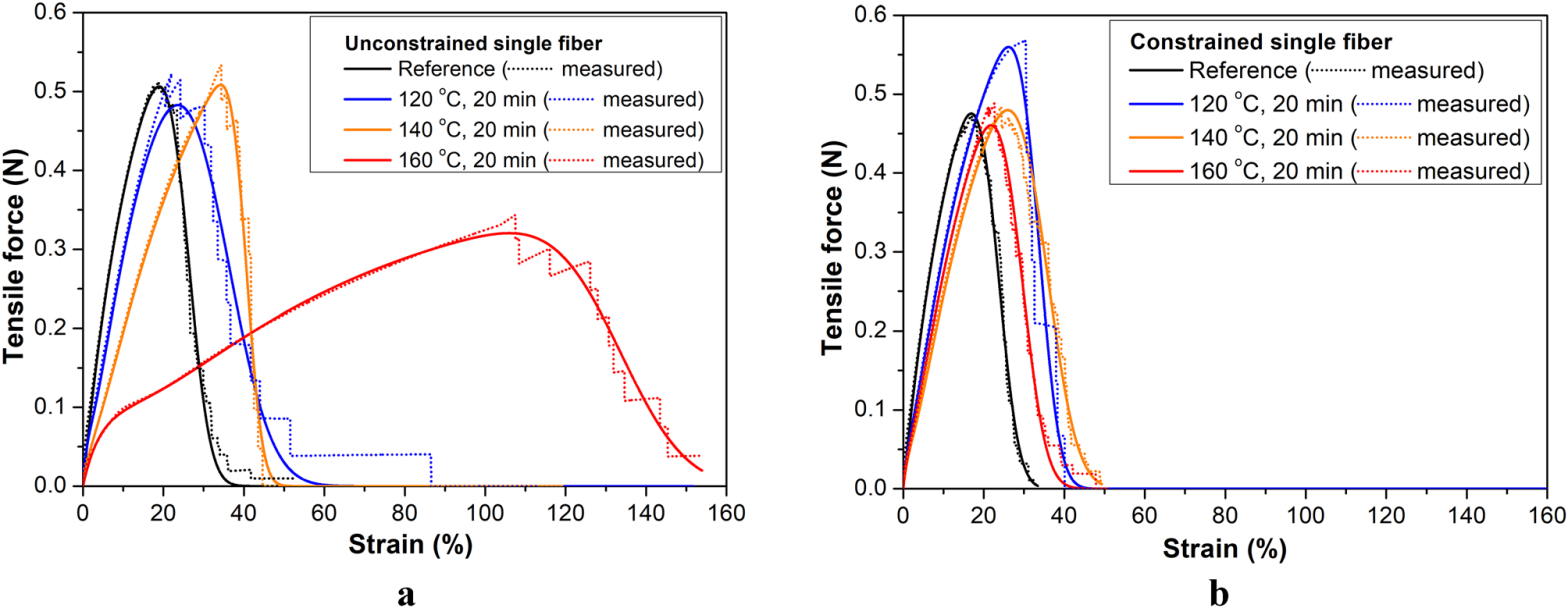
Measured and fitted E-bundle force–strain curves of fibers unconstrained (**a**) and constrained (**b**). Heat treatment was performed at different temperatures for 20 min.

**Figure 12:**
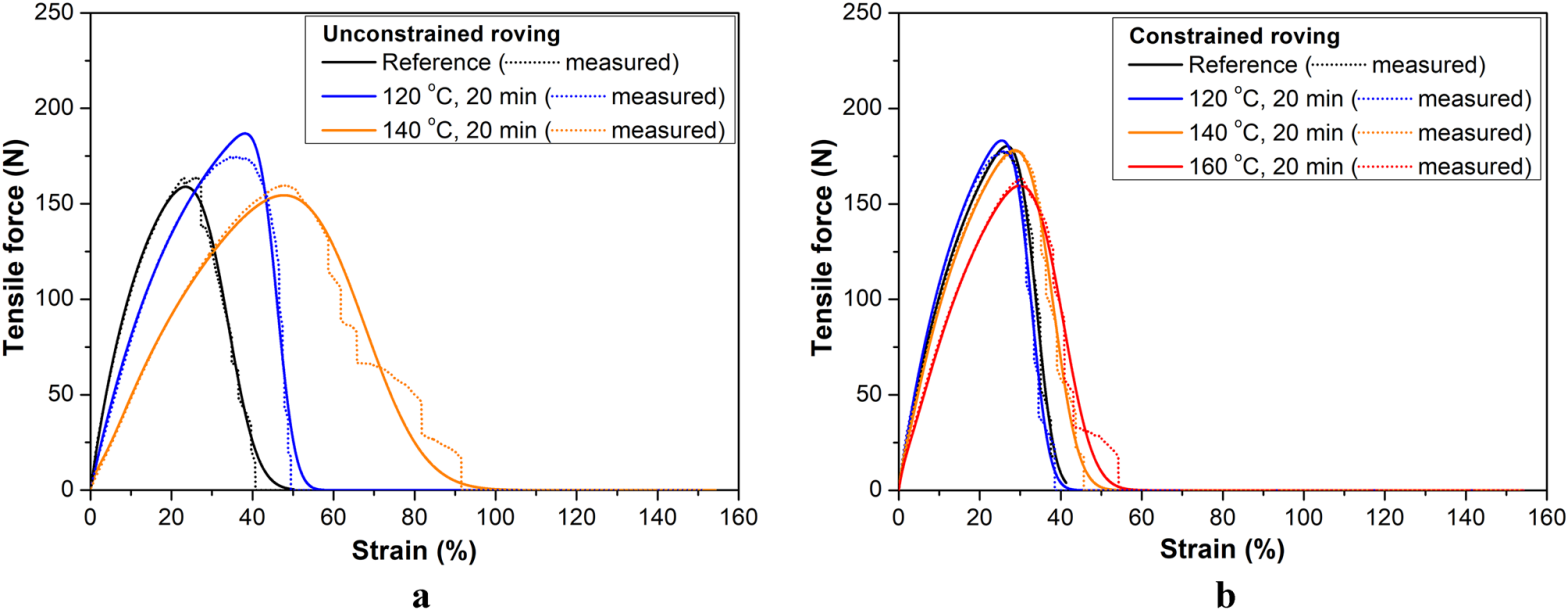
Measured and fitted E-bundle force–strain curves of rovings unconstrained (**a**) and constrained (**b**). Heat treatment was performed at different temperatures for 20 min.

Table 1 summarizes the results of fitting the response of the nonlinear E-bundles to the measured tensile force–strain curves obtained from tensile testing PP fiber and PP roving samples. The samples taken from the bobbins were constrained (T: samples pre-strained after taken from the bobbin) or were unconstrained (B: samples as taken from the bobbin) by winding and untreated (ambient temperature: T = 23 °C) or heat-treated at 120, 140, and 160 °C for 20 min. Figures 9 and 10 show the measured force–strain bundle curves (blue) of the single fibers and rovings untreated and heat-treated at 160 °C, which were calculated by averaging the single measurements point by point. Besides, they show the fitted force–strain curves of the FBC models, the tensile characteristic indicating undamaged functioning, and the reliability function describing the damage and the failure process. In Figs. 11 and 12, the fitted model curves together with the measured averages can be seen for the unstrained (B) and strained (T) single fibers (Fig. 11) and rovings (Fig. 12) untreated or heat-treated at different temperatures.

**Table 1:** Results of testing fibers and rovings and the fitting of FBC models.

		Tensile characteristics				Strain at break		E-bundle tensile curve					
	Temp. (°C)	a_0_ (N)	K_0_ (N)	a_A_ (N)	K_A_ (N)	MBS (%)	CV (%)	F* (N)	ε* (%)	FH* (%)	F_inf_ (N)	ε_inf_ (%)	ε_0.05_ (%)
Unconstrained, single fibers	23			0.70	5.25	26.00	16.15	0.51	18.92	84.32	0.27	26.48	33.40
	120	0.10	8.00	0.82	3.77	34.50	25.80	0.48	23.57	74.02	0.27	36.52	50.93
	140	0.10	3.50	0.81	2.43	40.50	7.65	0.51	34.22	89.22	0.27	40.75	45.86
	160	0.18	0.13	0.40	0.64	133.00	9.77	0.32	105.99	90.92	0.17	133.80	153.80
Unconstrained, rovings	23			200.00	1500.00	33.60	34.52	158.9	23.48	86.41	58.71	34.12	43.68
	120			240.00	984.00	45.90	7.82	2186.8	38.14	91.84	105.20	45.74	52.02
	140	20.00	1000.00	242.00	581.00	66.00	18.18	5154.48	47.75	80.31	484.86	67.93	87.55
Constrained, single fibers	23			0.70	5.25	23.30	17.17	0.48	16.90	82.24	0.26	23.84	30.42
	120	0.12	6.00	0.81	3.89	33.50	11.94	0.56	26.17	86.42	0.30	33.91	40.50
	140	0.08	4.80	0.82	3.12	35.00	17.14	0.48	25.97	79.54	0.26	36.03	45.85
	160	0.08	5.60	0.82	3.44	29.00	16.21	0.46	21.82	79.76	0.25	29.81	37.30
Constrained, rovings	23			240.00	1320.00	33.80	10.65	180.20	26.73	88.97	95.30	34.08	40.02
	120140	40.00	0.50	240.00	1440.00	32.50	11.05	5183.20	25.46	89.00	96.72	32.78	38.69
	160	40.00	0.35	240.00	1224.00	37.80	13.23	2178.10	28.64	86.86	95.16	38.28	46.51
				240.00	960.00	40.00	15.00	3159.67	29.98	83.36	86.59	40.77	50.63

We characterized the goodness of fitting the expected response of the nonlinear E-bundles with the relative mean squared error (RMSE) according to Eq. (11). It was below 5.5% (2.24–5.48%) in every case. In the case of testing 50 fibers, this error was 2.93–3.5%. When 8–12 fibers were tested, it was about 4.34–5.48%, where constraining did not have any significant effect. In the case of rovings, however, when the heat treatment temperature was not above 140 °C, the fitting error of the constrained rovings (T) (2.35–2.59%) was significantly smaller than that of the unconstrained rovings (B) (2.43–4.57%).

In addition to the fact that the fitted E-bundle formulas provide a usable mathematical description of the tensile force–strain relationship, their parameters in Table 1 make it possible to compare and analyze the changes in the expected mechanical behavior of the fibers or roving quantitatively, as the effect of the thermal treatment or the constraint. Among others, the parameters of the strain at break (mean and CV of ε_S_) determined through fitting the E-bundle as a model give more accurate information about failure than the parameters usually determined from the strain at force peak. Also, the distribution function Q_εS_(x) allows the calculation of the correct probabilities of failure at a given strain load, or confidence intervals for the estimated strength parameters of the reinforcing materials tested. The changes in these data numerically characterize the effect of the constraint applied and/or heat treatment, as shown in Table 1, where the latter represents the thermal effect during the production of the thermoplastic matrix composites reinforced by the fibers or roving examined.

According to the mean force–strain curves in Figs. 11a and 12a, the deformation properties of the unconstrained single fibers exhibit lower heat sensitivity than the unconstrained roving samples; moreover, their deformability is smaller also at room temperature. This can be attributed to the loose and/or oblique fibers. The heat treatment at 140 °C causes the deformability of both the fibers and the rovings to increase significantly in unconstrained samples (B), while the peak force (F*) of the B-fibers slightly changes. The heat treatment of the B-fibers at 160 °C (Fig. 11a), however, caused great changes in both their deformability and breaking force, which may be attributed to the intensive relaxation process. The tensile test could not be performed correctly on the B-roving at 160 °C because of its unstable behavior, as some fibers stuck locally due to the heat.

Figure 12a shows that the unconstrained rovings exhibited a significantly larger peak at 120 °C than the others at room temperature and 140 °C. In general, regarding the mechanical effects only, the peak value increases if the initial tensile stiffness and/or the mean breaking strain increases and/or the standard deviation decreases, hence the fiber bundle model can give a basis for analysis (see Eq. 6). The heat treatment at 120 °C reduced the initial tensile stiffness of untreated rovings (Table 1: from 1500 to 984 N) as a possible effect of the relaxation caused by the heat. At the same time, tensile strength characterized by the peak force (F*) significantly increased. Based on Eq. (6), the latter can be explained by the considerable increase in the mean breaking strain (MBS) (Table 1: from 33.6 to 45.9%) and the large decrease in the coefficient of variation (CV) (Table 1: from 34.5 to 7.8%). In the case of the heat treatment at 140 °C, the additional decrease in tensile stiffness (from 984 to 581 N) and the increase in CV (from 7.8 to 18.2%) was too large for the increasing MBS (from 45.9 to 66%) to compensate for. The strong relaxation induced by the heat obviously played an important role in this.

In the case of constrained fibers, the strain induced by winding resists shrinking caused by heat or may compensate for that to a certain degree and enable an increase in molecular orientation and crystallization. The constrained fibers heat-treated at 120 °C (Fig. 11b) exhibit a higher tensile force peak than the others and the mechanical explanation based on the fiber bundle model may be similar to the former one (see Table 1), but it should be completed with the effect of pre-straining and the heat-induced relaxation process during heat treatment. The diagrams show that the pre-strain generated by winding at room temperature (Figs. 11b and 12b) definitely inhibits intense relaxation, hence the formation of high heat sensitivity, thus it increases thermal stability. In addition, the mean values of fiber breaking strain (MBS) for the unconstrained and constrained fibers are significantly different only above 140 °C. If we consider the rovings, these mean values are more and more different starting from 120 °C, while at the same time, the coefficient of variation (CV) values are getting closer to each other.

The utilization factor (FH*) is determined by the CV of the fiber breaking strain when the fibers are straight and parallel to the direction of tensile load and their gripping is ideal. Every other statistical defect (crimping, obliquity, slippage from the grips) decreases FH*. Otherwise, thermal shrinking makes the unconstrained single fibers straight or at least less crimped; hence the utilization factor of the fibrous structure increases. In the other cases, this effect is valid only up to 120 °C (unconstrained roving and the constrained single fibers) because of the complex thermal and mechanical interactions.

In the case of rovings, the model fibers identified as the elements of the nonlinear E-bundle are not the same as the single fibers tested, since the model fibers represent the statistical structural defects in the rovings such as obliquity or crimping of the fibers. As opposed to that, the tensile test of the single fibers was performed in close to ideal circumstances regarding the orientation, crimping, and gripping of the fibers. Thus, in this case, the E-bundle fitted can be considered a bundle of fibers that are straight, parallel to the load direction, and gripped ideally. Otherwise, constraining the roving changes not only the mechanical state of the fibers but modifies their orientation and crimping as well. Consequently, the fitted E-bundle models enable additional comparisons between the unconstrained and constrained fibers and rovings.

The tensile stiffness of the second component in Eq. (2), K_A_, was applied and identified for every E-bundle model (Table 1), hence it can characterize the damageless behavior of the single fibers or the rovings at every temperature used. As expected, K_A_ significantly decreases with increasing temperature up to 140 °C. At 160 °C, which is relatively close to the melting temperature (171 °C), it becomes much smaller or larger than what the trend predicts for the unconstrained or constrained single fibers, respectively, indicating the reduction or preservation of the molecular orientation in the fibers. The constrained rovings do not show this phenomenon, which can be attributed to the statistical structural defects, such as oblique fiber orientation and crimping, as well as possibly not uniform gripping.

Equation (6) determines the relations between the characteristic strain values in Table 1, which are ε* ≤ E(ε_S_) ≤ ε_inf_ ≤ ε_0.05_, while their numerical value depends on the structural and thermomechanical state of fibers and rovings. At the strain level, ε_inf_, which is at the inflection point on the descending part of the expected force–strain curve, the damage rate is the highest. As expected, the mean breaking strain of model fibers increased with the temperature, meaning increasing deformability. Similar changes can be observed for the other characteristic strain values (Table 1). At the same time, the range of CV values of the fiber breaking strain was 5 and 6% for the constrained fibers and rovings, respectively, as opposed to 18 and 28% for unconstrained fibers and rovings, indicating the levelling effect of constraining.

As for the strength utilization factor (FH*) in Table 1, its variation with temperature was in a range of 17 or 12% for unconstrained fibers and rovings, while this range was only about half of that (6%) for constrained fibers and rovings, while the minimum value of F* slightly increased. This effect is due to constraining.

On the other hand, with the use of the strength utilization factor of the fibers and roving in ideal bundles, it is possible to predict fiber strength utilization in a real fiber bundle, which may be the model fiber reinforcement in a polymer composite, as shown in Table 2. Here, F_jS_ = k($\overline{\varepsilon }_{S}$) is a kind of mean breaking force of single fibers (j = f) or single rovings (j = r), which is given by F* and FH* in Eq. (10). The strength utilization factor of fibers in a real fibrous structure, such as a bundle of rovings can be predicted by FH* = F*/(n F_fS_) where F* is the peak force of the roving bundle related to one roving and n = 400 is the number of fibers in the roving.

**Table 2:** Strength utilization data for ideal fiber and roving bundle and its estimation for real fiber bundle.

Mechanical state during heat treatment	Temp. (°C)	Ideal bundle of fibers		Ideal bundle of rovings		Real fiber bundle
		FH* (%)	n*F_fS_ (N)	FH* (%)	F_rS_ (N)	FH* (%)
Unconstrained	23	84.3	241.9	86.4	183.9	65.7
	120	74.0	259.4	91.8	203.5	72
	140	89.2	228.7	80.3	192.4	67.6
	160	90.9	140.8	–	–	–
Constrained	23	82.2	233.5	89.0	202.6	77.2
	120	86.4	259.2	89.0	205.9	70.7
	140	79.5	241.4	86.9	205.1	73.8
	160	79.8	230.7	83.4	191.5	69.2

Table 2 shows that compared to the ideal bundle, the utilization factor for a real unconstrained fiber bundle is 18–22% lower than that of the ideal bundle while this difference for constrained fibers is only 5–16%, due to constraining. The exceptional case at 120 °C should be examined in a further study. Comparing the real utilization factors shows that constraining increased the range of variation from 66–72% to 71–77%.

This utilization factor may be used as a first assessment for a unidirectional reinforcement made of rovings in a polymer composite when the strength is estimated by a simple rule of mixture.

Consequently, constraining is a useful tool for preserving the reinforcing ability of PP fibers during manufacturing thermoplastic matrix composites.

## Conclusion

We studied the effect of heat treatment on the mechanical properties of high-tenacity polypropylene multifilaments and single fibers from them. We investigated the as-received rovings and the heat-treated fibers (at different temperatures for different periods) constrained (wound on an alumina plate) and unconstrained (free).

Using isothermal DMA tests, we pointed out that shrinkage becomes more significant near the melting temperature of the fibers (171 °C), and continuously increases at a given temperature but saturates after ca. 15 min at different values depending on treatment temperature. The residual tensile strength of the relaxed single fiber also dropped significantly at higher temperatures. We analyzed the statistical bundle behavior of the fibers and rovings and applied the simplest fiber-bundle-cell (FBC), the E-bundle, having fibers with nonlinear tensile characteristics.

From the tensile test results of the reference and the heat-treated (unconstrained and constrained) rovings and single fibers and their fiber bundle-based analysis, we can conclude that for unconstrained heat treatment, the tensile modulus and strength dropped markedly with increasing temperature and depended less on treatment time.

The tensile modulus of the constrained fibers treated for 5 min decreased less in the whole heat treatment temperature range but considerably decreased further with increasing treatment time. Conversely, their tensile strength decreased only slightly, and treatment time had a minor effect up to 180–190 °C (above the melting temperature of the fiber). The results proved that constraining is a useful tool for preserving the reinforcing ability of a high-tenacity polymer fiber. The constraining effect can be induced by loading the fibers, thereby preventing relaxation. The constraining of the fibers, multifilament, and tapes is possible by winding or fixing their ends on a frame or—a widely used method—applying pressure on the fibers (perpendicular to the fiber axis).

The PP fiber bundle models developed in this paper can make it possible to develop a material model for PP fiber-reinforced composites that may describe both deformation and the total damage and failure process.

## Data Availability

All data generated or analyzed during this study are included in this published article.
